# Quackery

**Published:** 1847-12

**Authors:** 


ARTICLE III.

### QUACKERY.

The attention that has been paid to this subject of late, and its importance, render it one of much interest. In a late number of the Journal, we noticed some legislative enactments in reference to the profession, and amongst other things, the law passed by the legislature of Maine for the suppression of nostrum vending, which required that all compound medicines sold in the State should be accompanied with a label, setting forth in full the ingredients of which they were composed, and the proportions of each, under certain penalties.

Here was a poser. But notwithstanding the shrewdness of Yankee law-makers, and their laudable designs to suppress the villanous practice of vending secret compounds as "king cure all," still the Yankee quacks soon found in the Chinese language a means of complying with the conditions of the law, without divulging their secrets to those not learned in that oriental tongue.

The legislature, finding, in their first attempt to suppress nostrum vending, that they had failed, instead of remedying the defects of the law foolishly repealed it; as much as to say "we give it up."

There was a principle involved in this law, or in its spirit, which we believe to be the true cure for the great traffic in nostrums. If the public knew the composition of the various patent and secret syrups, pills, electuaries, plasters, unguents, linaments, &c., &c., of which they annually in the United States consume millions of dollars worth—no, not *worth*, (for many of those bearing the highest price are intrinsically worthless,) but an amount which costs them millions, the trade would almost immediately stop, as it is the mystery that gives them currency, and completing the deception enables the quack to sell his aloes, that costs not to exceed 25 cents per pound, for at least ten times that amount, and molasses, worth 50 cents per gallon, when mixed with a little paregoric at one dollar per gill.

We shall hope to see the States generally try to protect the public, by requiring the composition of every medical compound dispensed within their limits, to be intelligibly set forth, so that when a patient takes physic, he may know something of the propriety of the bill he has been charged for it by his apothecary.

This, too, would be a good remedy for the nefarious practice we see alluded to by our cotemporaries, as practised between apothecaries and physicians on patients, in many of the larger cities of the United States, and, we fear, to a greater extent than is yet generally suspected, by which the physician receives a per centum on all prescriptions sent to the apothecary's shop, which influences him to prescribe larger quantities and frequently to change his prescriptions.

Now there is something so reasonable in this requisition, that few, if any, of the intelligent will fail to approbate it, unless interested against it. What, oppose the passage of a law that requires the salesman to tell the purchaser what it is he is selling to him ?

Nor would the public be particularly opposed to knowing what composes Brandreth's pills or Wistar's Balsam of Wild Cherry, et omnes genii, for they are not aware of the certain result—that it would rob them of almost all of their virtues.

The following from our esteemed correspondent A. W. Benton, M. D., contains some original suggestions on the subject, and we cheerfully insert them. E.

---

ANTIDOTE FOR QUACKERY.

Quackery as a cause, and gullibility as its effects, I believe have generally been treated by the faculty of well bred and honorable physicians with moral and intellectual remedies, with how much success the "thousand and one" advertisements in the newspapers, and parade of show bills and nicely labelled bottles will tell.

The cause, I consider entirely beyond the reach of moral remedies. When there is no conscience, moral remedies will have no effect. The *effects* of quackery I fear will seldom yield to intellectual remedies alone, but if backed up by proper physical means it is possible they might succeed. I have long been of the opinion that some means might be devised to lessen, if not entirely suppress the sale of the ten thousand quack medicines now offered for sale in every store, tavern, grocery, and doggery throughout the length and breadth of our land. People will have some domestic remedy to apply in cases of slight indisposition, rather than send for a doctor for every trifling ailment, and I am not sure but it is right they should have. The only apology I know of for the quack is, that the community is not supplied with medicine from any other source.

The plan I would suggest is to put in operation such measures as will supply the community with a sufficient number of some of the cheapest and safest medicines, (either officinal preparations or other compounds with the ℞ attached,)to fill the place of the quack medicines.

With a modest advertisement, setting forth their value, stating that they are the remedies approved by the most eminent medical men in both Europe and America, and laid down in the standard works on medicine. Also stating that if these medicines fail to cure, and they are compelled to send for a physician they have the advantage of the Dr's. knowing what they have been taking, and consequently is better enabled to know what medicine to give next; whereas if they use quack medicine, he does not always know whether the patient is sick in consequence of the medicine taken, or something else.

With such a recommend would they not compete with the quack nostrums now in vogue? And would it not be well for every Medical Society, to put in operation such means as would supply the district of country over which their influence extends, with a well selected number of common remedies in sufficient quantity to meet the demand? We know that many of the quacks amass princely fortunes from the profits of their sales, and it does seem to me, that the community might be supplied with medicines of known efficacy without a hazard of life.

I would suggest this plan for reflection, to be acted on as judgment, matured by deliberation, may dictate.

Sterling, Jan. 1848. A. W. BENTON.

